# Substance specific EEG patterns in mice undergoing slow anesthesia induction

**DOI:** 10.1186/s12871-024-02552-3

**Published:** 2024-05-03

**Authors:** David P. Obert, David Killing, Tom Happe, Philipp Tamas, Alp Altunkaya, Srdjan Z. Dragovic, Matthias Kreuzer, Gerhard Schneider, Thomas Fenzl

**Affiliations:** 1 School of Medicine and Health, Department of Anesthesiology and Intensive Care, Technical University of Munich, 81675 Munich, Germany; 2 Department of Anesthesia, Critical Care, and Pain Medicine, Massachusetts’s General Hospital, Boston, MA 02114 USA; 3 Harvard Medical School, Boston, MA 02115 USA

**Keywords:** EEG, Sevoflurane, Ketamine, Dexmedetomidine, Propofol, Murine model

## Abstract

The exact mechanisms and the neural circuits involved in anesthesia induced unconsciousness are still not fully understood. To elucidate them valid animal models are necessary. Since the most commonly used species in neuroscience are mice, we established a murine model for commonly used anesthetics/sedatives and evaluated the epidural electroencephalographic (EEG) patterns during slow anesthesia induction and emergence. Forty-four mice underwent surgery in which we inserted a central venous catheter and implanted nine intracranial electrodes above the prefrontal, motor, sensory, and visual cortex. After at least one week of recovery, mice were anesthetized either by inhalational sevoflurane or intravenous propofol, ketamine, or dexmedetomidine. We evaluated the loss and return of righting reflex (LORR/RORR) and recorded the electrocorticogram. For spectral analysis we focused on the prefrontal and visual cortex. In addition to analyzing the power spectral density at specific time points we evaluated the changes in the spectral power distribution longitudinally. The median time to LORR after start anesthesia ranged from 1080 [1st quartile: 960; 3rd quartile: 1080]s under sevoflurane anesthesia to 1541 [1455; 1890]s with ketamine. Around LORR sevoflurane as well as propofol induced a decrease in the theta/alpha band and an increase in the beta/gamma band. Dexmedetomidine infusion resulted in a shift towards lower frequencies with an increase in the delta range. Ketamine induced stronger activity in the higher frequencies. Our results showed substance-specific changes in EEG patterns during slow anesthesia induction. These patterns were partially identical to previous observations in humans, but also included significant differences, especially in the low frequencies. Our study emphasizes strengths and limitations of murine models in neuroscience and provides an important basis for future studies investigating complex neurophysiological mechanisms.

**Supplementary Information:**

The online version contains supplementary material available at 10.1186/s12871-024-02552-3.

## Introduction

General anesthesia is administered to a million people every day [1]. This number underlines the relevance of anesthetic agents. Despite enormous research efforts and a history of more than 150 years the exact mechanisms of how these drugs induce unconsciousness are still not fully understood [2, 3]. Even though the molecular targets have been identified, the neurol circuits involved in anesthesia-induced loss of responsiveness remain elusive [4]. Much of the knowledge which has been collected over the last decades and the main organism in neuroscience to study anesthesia-mediated effects is the mouse [5].

A widely established method to evaluate the electrophysiological effects of anesthetics on the brain is the electroencephalogram (EEG), which has first been described in 1929 [6–8]. As it is a non-invasive technique, it can easily be applied and allows the analysis of cortical and to a limited degree also of subcortical activities [9]. In the last decades, the EEG signatures of the most commonly used anesthetics/sedatives have been described in humans in more details [10–12]. The volatile anesthetic sevoflurane – primarily mediating its effect through γ-aminobutyric acid (GABA), glycine, acetylcholine, and glutamate receptors [13] – was shown to induce frontal alpha and delta oscillations [10]. These features were shared with the intravenously administered drug propofol [10, 14]. The main mechanism of action of propofol is the enhancement of the GABA_A_ receptor function by binding to its β subunit but it also modulates the activity of acetylcholine and glycine receptors [15]. In contrast to propofol, sevoflurane also exhibited a distinct theta signature [10]. The EEG signature of the α_2_-agonist dexmedetomidine was characterized by an increase of the power in the slow frequencies (< 8 Hz) and a decrease in the faster frequencies (> 20 Hz) [16]. In addition, dexmedetomidine induced spindles, similar to the ones observed during sleep [16]. An increased delta power was also found after the administration of ketamine [11]. Ketamine has an almost unique pharmacodynamic profile as it induces analgesia, amnesia, loss of consciousness, and immobility [17]. Its mechanism of action is highly complex including neuromodulation, gene expression, cellular effects and alteration of channel function, e.g. blockade of the N-Methyl-D-aspartate (NMDA) receptor [18]. The ketamine induced EEG pattern was not only characterized by a strong delta activity, but this strong delta activity was interrupted by gamma bursts, which presented a unique feature of ketamine. Furthermore, a persistent beta activation under ketamine induced anesthesia was described [19]. The knowledge about these EEG signatures is also used clinically to evaluate the hypnotic component of general anesthesia [20, 21].

However, superficial electrophysiological recordings only partially reflect subcortical activity, for which reason animal models are needed to further elucidate the neurol circuits involved in anesthetic-induced unconsciousness [22]. The present study was designed to develop standardized face validity and constructive validity in mice for the major anesthetics dexmedetomidine, ketamine, propofol and sevoflurane as a common prerequisite for neurobiological research. It was based on slow anesthesia inductions allowing to detect transient changes in the brain activity.

## Materials and methods

### Animals

All experimental procedures were approved by the Commission on Animal Health and Care of the State of Upper Bavaria, Germany (ROB-55.2–2532.Vet_02–19–121). Reporting of the animal research in this study complies with the ARRIVE guidelines and animals were treated in accordance with recommendations in the Guide for the Care and Use of Laboratory Animals [23, 24]. Adult male mice (C57BL6\N, 11–16 weeks, 23–32 g, *n* = 44, Charles River Laboratories, Germany) were included in the study. Animals were housed individually, but with acoustic, visual and olfactory contact and *ad libitum* access to food and water [25, 26]. A 12 h light-dark cycle (light on: 9am, light off: 9pm) was maintained. All experiments were performed during the light ON cycle. Since previous studies showed an influence of the estrous cycle on sleep behavior in female mice with a potential impact on basal EEG parameters, we exclusively used male mice [27].

### Surgery

The surgical procedure depended on the group assignment. All animals receiving dexmedetomidine, propofol or ketamine received a central venous catheter (CVA) for drug application and were treated surgically as previously described [26, 28–30]. The animals with sevoflurane treatment did not undergo a CVA implantation.

Briefly, for anesthesia induction animals were placed in an acrylic glass box (custom made) with 4 Vol.-% isoflurane (CP Pharma, Germany). After loss of righting reflex (LORR) the mice were transferred to a stereotactic frame (Leica Mikrosysteme Vertrieb GmbH, Germany) and anesthesia was maintained (1.6-2 Vol% isoflurane, flow rate: 190 ml/min, Univentor 410, AgnTho’s, Lidingö, Sweden). Analgesia was provided through a subcutaneous injection of carprofen (Zoetis, Germany) after anesthesia induction (5 mg/kg BW). The animals were placed on a homeothermic monitoring system (Harvard Apparatus, USA) to maintain body temperature at 37 °C. After shaving the head and neck area, a small incision was made in the neck to provide an exit for the CVA. Mice were then transferred to a supine position and hair was removed in the right parasternal and neck area followed by an approx. 1 cm long skin incision. The connective tissue and the right jugular vein were carefully prepared. Inflows to the jugular vein as well as the cranial part of the vein itself were ligated [28]. A venotomy was performed using micro-scissors (#OC497R, Aesculap AG, Germany) and the catheter (Becton Dickinson and Company, USA) was inserted into the vein. To ensure correct placement, the catheter was checked for heart-rate-synchronous pulsation and aspiration. Afterwards, the catheter was secured into place using silk sutures (Johnson & Johnson, USA) and tissue adhesive (Br Braun Surgical S.A., Spain) and tunneled subcutaneously to the neck incision. The animals were transferred back to the stereotactic frame, the neck incision was extended, and connective tissue and periosteum were removed. The drilling spots for the epidural EEG electrodes were marked stereotactically and craniotomies were performed using a dental micro driller (Ø 600 μm, Henry Schein Dental, Germany). The electrodes were placed above the prefrontal (PFC; 3.1 mm anterior to bregma, 1.5 mm lateral to midline), primary motor (MC; 1.5 mm anterior to bregma, 2 mm lateral to midline), sensory (SC; 0.9 mm posterior to bregma, 3 mm lateral to midline), and visual cortex (VC; 2.9 mm posterior to bregma, 2.3 mm lateral to midline) [31]. Additional holes were added to insert a ground electrode (frontal) and two jeweler’s screws for stability (parietal). A printed circuit board socket (Preci-Dip, Switzerland) holding the electrodes was then fixed to the mice’s head using dental cement (Kulzer GmbH, Germany). The construction was sealed off using dental cement providing stability and isolation for each individual wire. After surgery, the animals were placed back in their home cage and an electrically shielded recording cable was connected to the electrode array. The cable was connected to a weight neutral swivel system (custom made, Streicher M., Innsbruck, Austria) and a commutator (model SL-20, DRAGONFLY, USA), allowing the animal unrestricted movement in its home cage. All animals were given a ten-day recovery period before experimental anesthesia was performed. Analgesia was ensured through carprofen (Zoetis, Germany) added to the animals’ drinking water (0.067 mg/ml) for three postsurgical days.

### Experimental protocol

Eight mice were included for each anesthetic. Anesthesia protocols had to be adjusted to substance specific characteristics to accommodate for distinct mechanisms of action, behavioral effects, and animal tolerance. Each experimental anesthesia was preceded by 30 min of baseline (wake) EEG recording and followed by at least another 30 min of post-Return of Righting Reflex (RORR) recording. For each substance, oxygen saturation (MouseOx Plus, Starr Life Science corp., USA) and body temperature during anesthesia was measured and maintained (O_2_ > 92%, T at 37.0 °C).

### Sevoflurane anesthesia

The animals were placed in a transparent, hermetically sealed acrylic glass chamber equipped with a heating pad to prevent hypothermia (custom made, volume of 7.1 l). Inspiratory sevoflurane (CP Pharma, Germany) concentration was measured using a Capnomac Ultima monitor (Datex-Ohmeda Inc., Madison, USA). The fresh gas flow was 1.5 l/min, with an inspiratory oxygen concentration of 0.5 throughout the experimental procedure. Anesthesia started at an inspiratory concentration of 0.2 Vol.-% and was increased by 0.2 Vol-% every two minutes through loss of righting reflex (LORR) until 30 consecutive seconds of suppression in the online EEG could be observed. The inspiratory concentration of sevoflurane was then decreased by 0.2 Vol.-% every two minutes until zero.

### Propofol anesthesia

To keep the volume load to a minimum, propofol was applied at 2% (20 mg/ml, Fresenius Kabi Deutschland GmbH, Germany). A 1 ml perfusor syringe (Hamilton Bonaduz AG, Switzerland) was filled with propofol, mounted into a microinjection pump (Carnegie Medicin, Sweden), and connected to the catheter using a perfusor line (B. Braun Melsungen AG, Germany). The catheter was then flushed with 25 µl propofol. This was followed by a 30-minute waiting period to ensure that any amount of propofol which might have been injected during the flushing procedure had worn off. Anesthesia started at a flow rate of 0.5 µl/min which was increased by 0.5 µl/min every two minutes until LORR occurred, then kept at that respective flow rate for five minutes before being stopped. Deviation from a 30 s suppression protocol was necessary since the mice would not tolerate the corresponding amounts of propofol.

### Ketamine anesthesia

For introducing anesthesia, 25 mg/ml of ketamine (CP Pharma, Germany) were used. All further steps for the maintenance of the experimental anesthesia were performed as described in the propofol protocol. As ketamine only induces burst suppression during GABAergic anesthesia or at very high doses which were not tolerated by the animals, we could not apply the sevoflurane protocol [32].

### Dexmedetomidine anesthesia

Since dexmedetomidine (Vetpharma Animal Health, S.L., Spain) is rather a sedative and a perioperative adjuvant than an anesthetic, the occurrence of burst suppression could not be used as an electrophysiological target parameter [33]. A dose/response trial revealed that a weight-adapted dose of 0.45 µg/g body weight (BW) induced a prolonged LORR in all animals which we defined as a behavioral endpoint After connecting the syringe pump to the catheter, the catheter was flushed with 25 µl of dexmedetomidine solution and 30 min later the infusion was started at a flow rate of 1 µl/min with a concentration of 0.5 µg/µl. Flow rate was increased by 1 µl/min and stopped once the weight-adapted target dosage had been reached. Throughout the procedure the animals were video recorded and RORR was noted.

### Data acquisition

The EEG channels were individually pre-amplified with a headstage (1x amp.), followed by differential amplifiers for each signal (1000x amp. band-pass: 0.1–100 Hz, DPA-2FL, npi electronic, Germany) and sampled at 250 Hz (Power1401–3 with Spike2, Cambridge Electronic Design Ltd., UK). Raw signals were imported to MATLAB 2021b (The Mathworks, Natick, USA) for further analyses.

### EEG processing

For analyses the MATLAB toolbox *eeglab* was used [34]. Firstly, the data were high pass filtered at 0.5 Hz and low pass filtered at 47 Hz (50 Hz line noise elimination) using the *eeglab* function *eegfilt*. Artifacts were automatically removed with the *eeglab* function *pop_clean_rawdata* utilizing the Artifact Subspace Reconstruction (ASR) with the ASR value set to 20 as recommended for automated scripts [35]. Artifacts were rejected and missing EEG periods were not interpolated. The power spectral densities (PSD) were calculated using the pwelch function (NFFT = 512, window length = 10 s, shift = 1 s) to calculate density spectral arrays (DSA). As the time period from the start of administration of the anesthetic drug to LORR was not constant in all mice, the interval had to be normalized in order to be comparable between animals. We divided for each mouse the induction period (start anesthesia – LORR) into 100 steps and calculated the median PSD for each step. For dexmedetomidine we defined the LORR which was followed by a prolonged period of LORR (> 30 min) as the end of the induction period since we were interested in general anesthesia (non-arousable) rather than sedation (arousable).

In humans the frequency range 1–30 Hz is rather arbitrarily divided into delta, theta, alpha, beta, and gamma bands. Even the International Federation of Clinical Neurophysiology included various definitions of the frequency bands in its recommendation [36]. For this reason, we performed a continuous analysis of the spectrum for statistical analysis and used the nomenclature of frequency bands for descriptive purposes. In the present study we defined the frequency bands as delta 0.5–4 Hz, theta 4–8 Hz, alpha 8–13 Hz, beta 13–32 Hz, and gamma > 32 Hz.

### EEG spindle detection

Spindle (SP) detection used an automated MATLAB-based method [37]. Artifacts in raw EEG traces were corrected using the *eeglab* function pop_clean_rawdata (ASR = 20). Traces were bandpass filtered (10–15 Hz) based on mice’s mean peak SP frequency (11 Hz) [38].Root mean square (RMS) of filtered EEG was computed (750 ms window), and RMS values cubed. A two-threshold approach established SP detection criteria. Detection parameters: minimum SP duration: 0.5 s, maximum SP duration: 2 s, inter-SP interval: 0.1 s. SP density (SPs/min) and distribution were analyzed for all anesthetic agents. The empirical cumulative distribution of SP time points and slope between neighboring SP pairs were calculated. Slope values were smoothed (MATLAB’s smooth, span = 100) and interpolated to 1000 points (interp1, modified Akima interpolation) [39]. The median of each mouse’s data visualized anesthesia-dependent median SP distribution.

### Statistical analyses

Due to the small sample size, we assumed a non-parametric testing. Data are presented as median [1st quartile; 3rd quartile]. Statistical significance was defined as *p* < 0.05. The EEG for the baseline PSD were chosen from the end of the baseline period. The EEG was visually inspected and an artifact-free interval was selected. For the rest of the PSDs an interval of 20 s (± 10 s) was chosen around the given point in time (i.e., LORR, RORR + 120s etc.). The resulting median PSD vector was calculated by means of the median and 1st and 3rd quartile power values for each frequency in the chosen interval. To assess differences between the PSDs at the given intervals we used an effect size: the area under the receiver operating characteristics curve (AUC). To evaluate differences the AUC compares the ranks of the individual data points of two data sets. We corrected for multiple comparison by using a “cluster-based” approach and only mentioned findings in the results if at least two neighboring frequencies showed significant or relevant effects. The AUC calculation with 10k-bootstrapped 95% confidence intervals (CI) was done using the MATLAB-based MES toolbox [40]. An AUC of 0.5 is indicative of “no effect” and an AUC > 0.7 or AUC < 0.3 indicates a relevant effect [41]. If the 95% CI did not include 0.5, the result was considered significant. This approach has previously been applied to evaluate changes in band powers [42, 43].

## Results

### Induction of anesthesia

In total 12 animals had to be excluded from the analysis. Two animals died during the surgery, two animals died during experimental anesthesia, four animals had to be excluded due to catheter dislocation and the data of four animals could not be analyzed due to amplifier malfunction and technical issues.

**Fig. 1:**
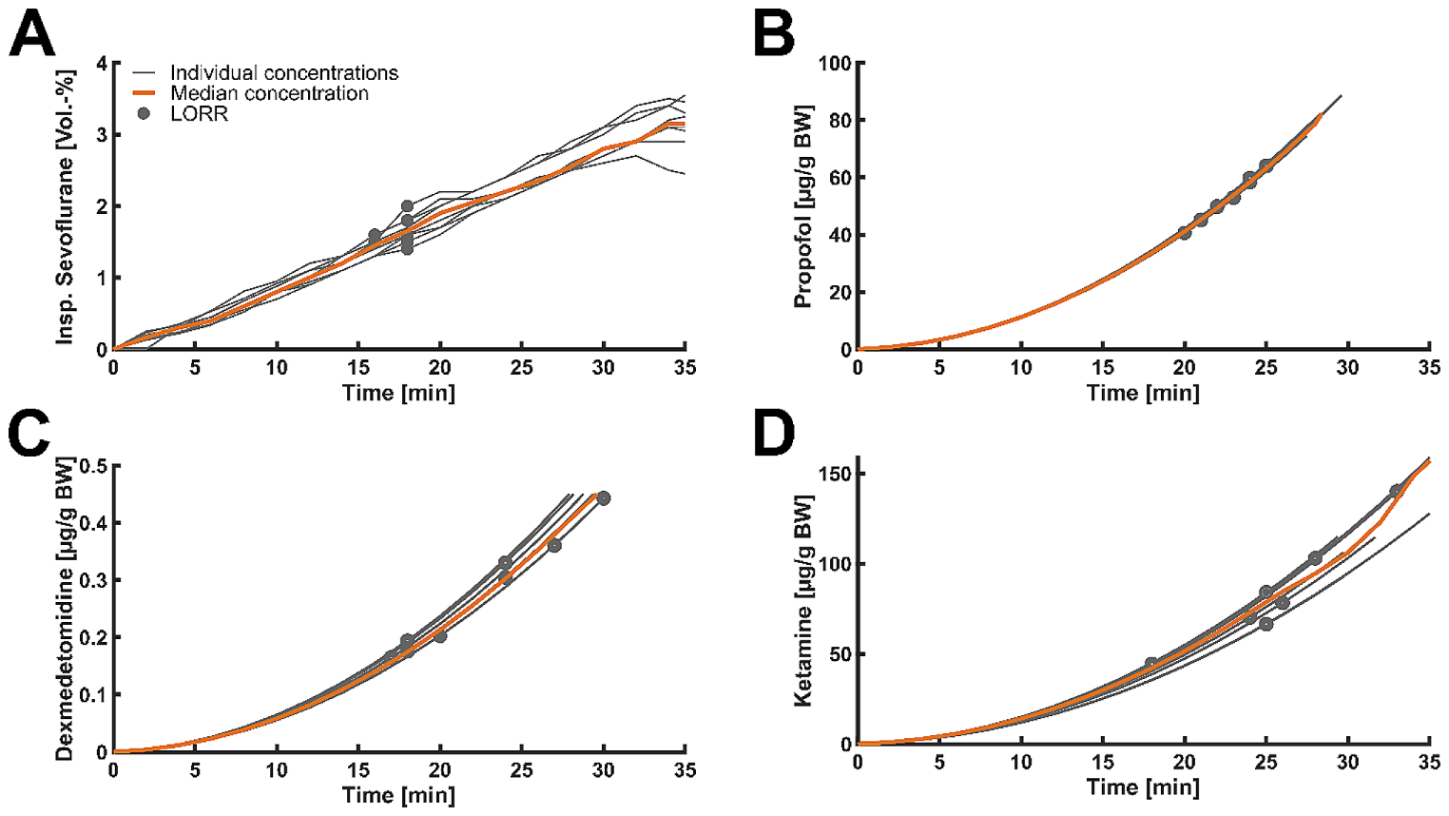
Graphs showing individual (gray lines) as well as median (orange lines) anesthetic concentrations over time during induction;circles indicate LORR. BW: body weight

In the sevoflurane group three animals were excluded from analysis. During slow sevoflurane induction mice lost righting reflex approximately 1080 [960; 1080] s after start of anesthesia and at an inspiratory sevoflurane concentration of 1.6 [1.5; 1.6] Vol.-%. RORR occurred 2520 [2220; 2700] s after LORR. The median inspiratory sevoflurane concentration at RORR was 1.2 [0.85; 1.3] Vol.-% (Fig. 1-A).

Animals undergoing propofol anesthesia lost righting reflex 1354 [1245, 1432] s after the start of induction at a median dosage of 2.58 [2.19, 2.92] µg/g BW. RORR occurred 716 [501, 894.5] s after LORR (Fig. 1-B).

For dexmedetomidine, 75% of mice went through multiple cycles of loss and return of righting reflex. The first LORR occurred at a median of 1313 [1088; 1537] s after the start of induction at a dosage of 0.272 [0.1955; 0.3485] µg/g BW. A consistent state of LORR was reached 1706 [1416; 1995] s after the start of anesthesia. The median duration of this vigilance state was 6502 [5028; 7976] s. A dosage of 0.432 [0.371; 0.45] µg/g BW was applied until occurrence of final LORR (Fig. 1-C).

In the ketamine group, the animals lost righting reflex 1541 [1455; 1890] s after the start of induction at a dosage of 78.6 [67.9; 126.2] µg/g BW. Righting reflex was lost for 553 [486; 761] s (Fig. 1-D).

### Baseline vs. anesthesia induction

**Fig. 2:**
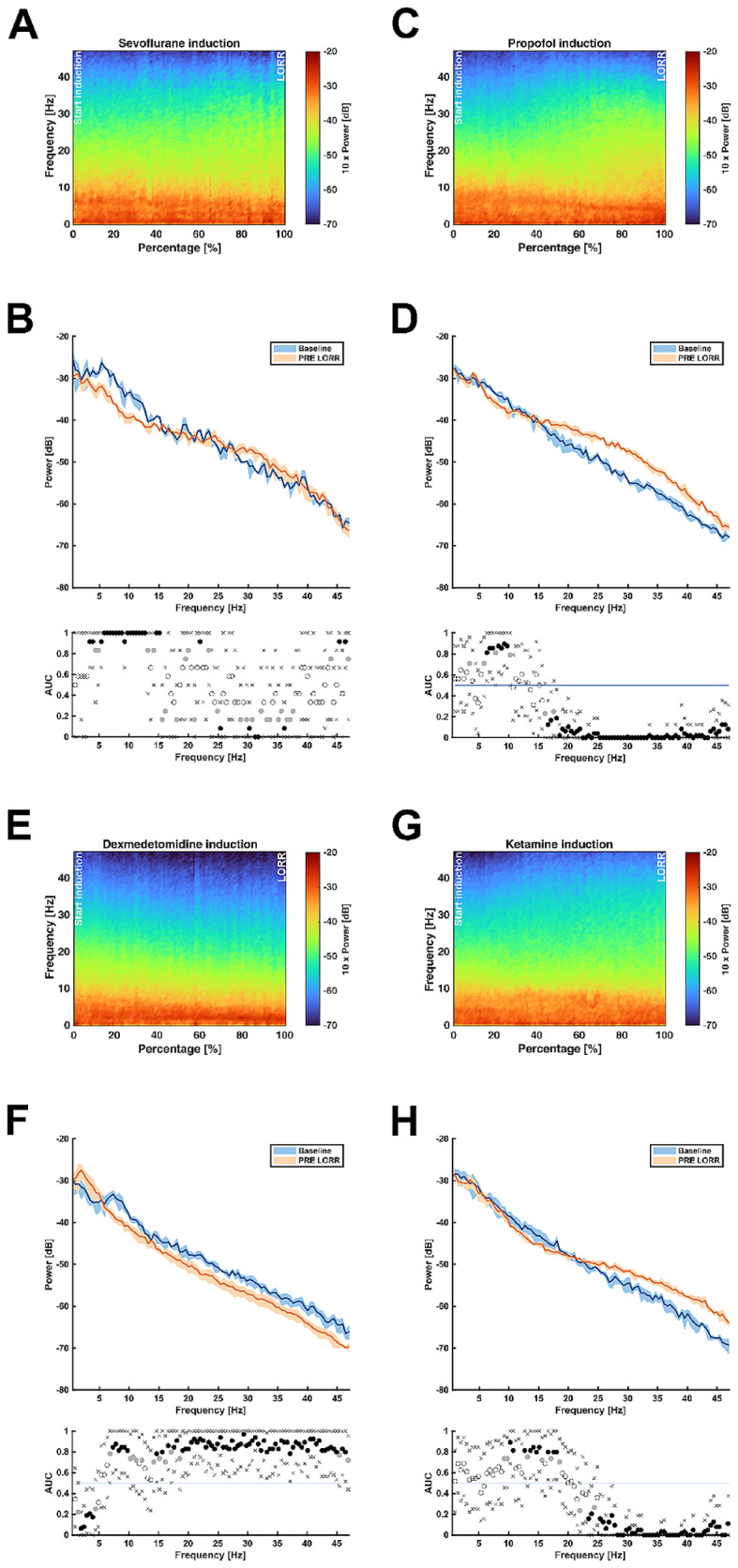
**A**, **C**, **E**, **G**: Median density spectral arrays (DSA) from left visual cortices (VC) showing the slow induction phase with all four anesthetics; **A** = sevoflurane, **C** = propofol, **E** = dexmedetomidine, **G** = ketamine; x-axis shows normalized time from start of induction (0%) to loss of righting reflex (LORR) (100%) in percentage. **B, D, F, H:** Median power spectral density arrays (PSD) from left VCs illustrating absolute power distribution during baseline and pre-LORR (85–95% from start of induction to LORR) intervals; **B** = sevoflurane, **D** = propofol, **F** = dexmedetomidine, **H** = ketamine; blue graph showing baseline spectrum, orange graph showing PRE LORR spectrum; shaded areas indicating 95% confidence intervals; *n* = 8; area under the curve (AUC) showing statistical significance of differences between power spectra, black dot indicating significant difference, gray dot indicating relevant effect, white dots indicating no statistical significance

The DSA of the induction phase of sevoflurane anesthesia showed subtle changes in the VC with a decrease in the theta and delta frequency band (Fig. 2-A). Comparing the spectral composition at start of anesthesia and right before LORR, a significant decrease in the delta (3.4–3.9 Hz), theta (at 5.4–8 Hz) as well as alpha band (8–12.7 Hz) was found. There was also a significant decrease in the low-beta power (14.6–15.1 Hz), while the activity in the high-beta (29.8–32 Hz) and gamma band increased (32–37.6 Hz) (Fig. 2-B). Propofol induced a decrease in the theta (5.9–8 Hz) and alpha band (8–10.3 Hz). On the other hand, the PSD demonstrated a significant increase in the beta (16.1–32 Hz) and gamma band (> 32 Hz) (Fig. 2-C and -D). During dexmedetomidine anesthesia, a continuous shift towards slower frequencies representing a general slowing of the EEG in the VC was recorded (Fig. 2-E). The power in the gamma (> 32 Hz), beta (14.1–32 Hz), alpha (8–11.2 Hz) and theta band range (6.8–8 Hz) decreased significantly, whereas the delta band (1.5–4 Hz) power increased significantly (Fig. 2-F). The DSA deriving from VC during ketamine application showed an increase in higher frequency ranges (> 25 Hz) (Fig. 2-G). The corresponding PSD demonstrated a significant increase especially in the high-beta (at 23.4–24.4 and 25.3–32 Hz) and gamma band (> 32 Hz). In the alpha (9.3–10.7 Hz) and low-beta band (12.7–15.6 and 16.6–18.6 Hz) a decrease was observed (Fig. 2-H).

### Baseline vs. anesthesia maintenance

**Fig. 3:**
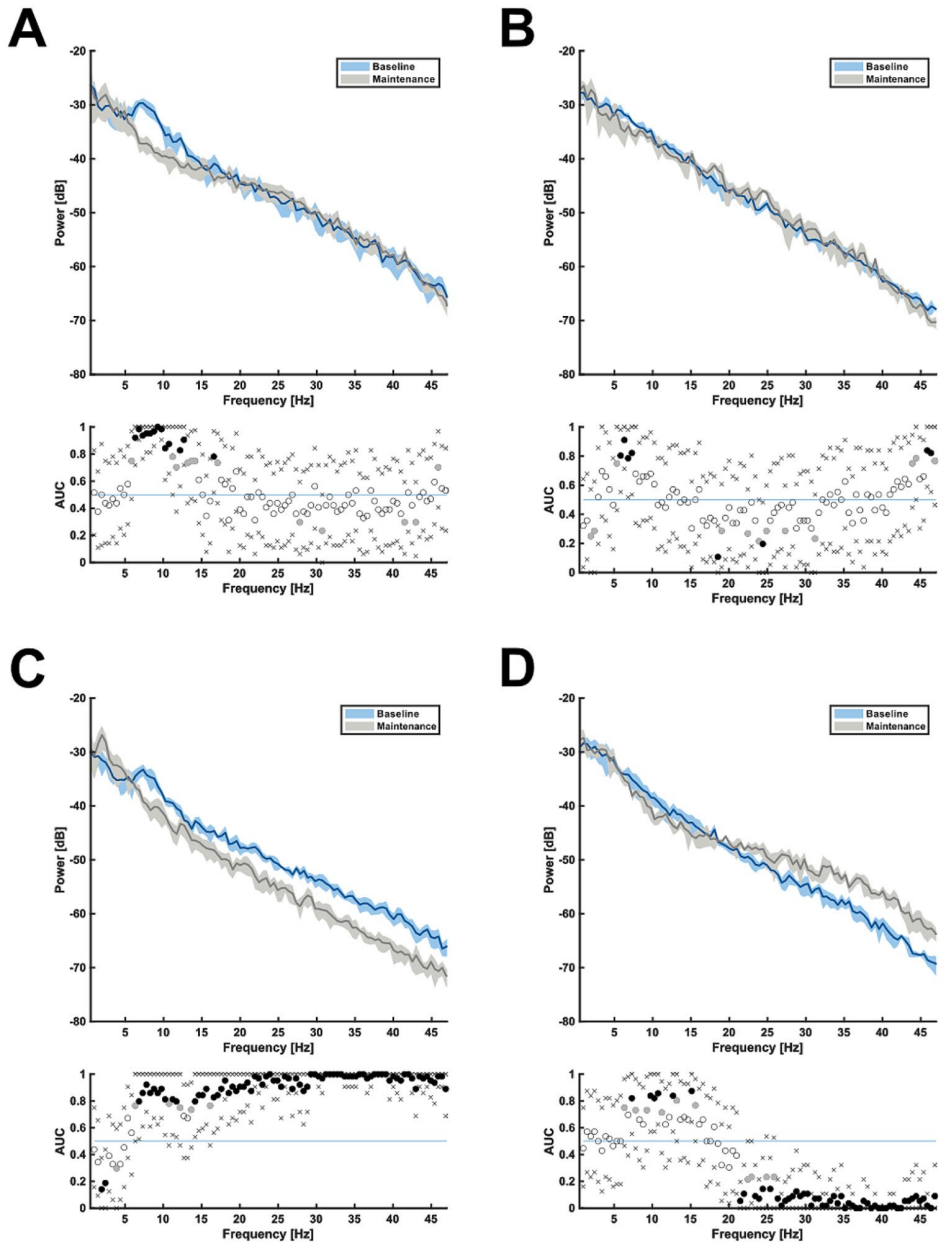
Median PSD arrays from left VC illustrating the absolute power distribution during baseline and anesthesia maintenance; **A** = sevoflurane, **B** = propofol, **C** = dexmedetomidine, **D** = ketamine; blue graphs: baseline spectrum; gray graph: burst suppression adjusted maintenance spectrum; shaded areas indicating 95% confidence intervals

A comparison of VC recordings at baseline versus anesthesia maintenances revealed a significant decrease in the theta (5.9–8 Hz), alpha (8–12 Hz), and low-beta band (12–14.2 Hz) for sevoflurane. The PRE LORR increase in the beta band and gamma band was not present anymore (Fig. 3-A). Propofol induced a reduction in theta power (5.4–7.3 Hz) and a reversal of the broad activation in frequencies above 16 Hz, as seen in the comparison between wake and PRE LORR (Fig. 3-B). The analysis of the recordings originated from the PFC demonstrated a significant increase in alpha power when compared to baseline (Supp. Figure 1B). Dexmedetomidine anesthesia revealed a high power in the delta band with a reduction of all frequencies higher than 6.4 Hz (Fig. 3-C). During anesthesia maintenance with ketamine, a decrease in the low-beta (12.7–13.2 and 15.1–15.6 Hz) and alpha band (9.3–11.2 Hz) was observed. The power in the beta band (21.5–32 Hz) and gamma band (> 32 Hz) increased (Fig. 3-D).

### Anesthesia maintenance vs. emergence

**Fig. 4:**
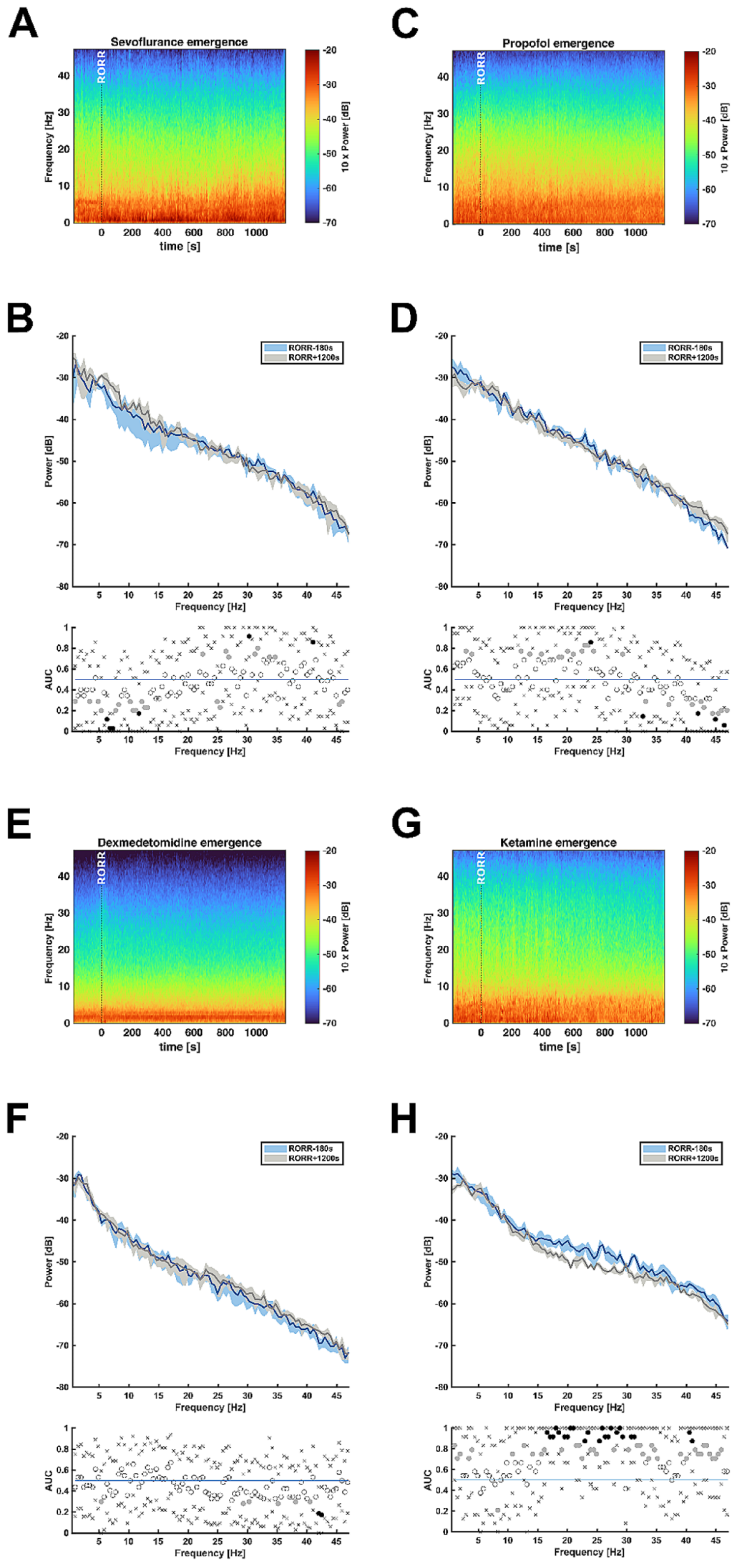
**A**, **C**, **E**, **G**: Median DSAs from left VC showing emergence from anesthesia; **A** = sevoflurane, **C** = propofol, **E** = dexmedetomidine, **G** = ketamine; x-axis shows time in seconds with 0 indicating RORR which is also marked with a dashed line. **B**, **D**, **F**, **H**: Median PSD arrays from left VC illustrating absolute power distribution during anesthesia maintenance (RORR-180 s) and emergence (RORR + 1200 s); **B** = sevoflurane, **D** = propofol, **F** = dexmedetomidine, **H** = ketamine; blue line represents the spectrum at RORR-180 s and gray line at RORR + 1200 s; shaded areas indicating 95% confidence intervals

During emergence from anesthesia spectral trajectories for all substances showed a reversal of the effects seen during anesthesia induction. Sevoflurane elicited an increase in absolute theta power (6.3–8 Hz) and alpha power (10.3–12 Hz), in contrast to the corresponding decrease during anesthesia induction (Fig. 4-A, -B). During emergence from propofol anesthesia power in the gamma band significantly increased. (Fig. 4-C, -D). Dexmedetomidine showed minimal spectral changes when comparing the EEG at 120s before RORR to RORR + 1200 s (Fig. 4-E, -F). However, when analyzing the hours after RORR, the power in the higher frequencies slowly increased and 10 h after RORR the spectral composition resembled the one at baseline (Supp. Figure 2). Mice treated with ketamine showed a reversal of spectral composure with broad decreases in the beta band and gamma band (Fig. 4-G, -H).

### Spindle analysis

During anesthesia induction, no spindle-specific differences between individual substances were found (dexmedetomidine: 1.52 SP/min [1.07; 1,64]; propofol: 0.71 SP/min [0.51; 1.69]; ketamine: 1.55 SP/min [1.11; 1.81]). During sevoflurane induction, no spindles were detected. During the first half of anesthesia maintenance, the number of spindles increased significantly under dexmedetomidine anesthesia to 2.48 SP/min [2.12; 2.68], but not for propofol: 1.06 SP/min [0.88; 1.65] and ketamine: 1.30 SP/min [1.13; 1.64]. Spindle distribution analysis during maintenance of dexmedetomidine anesthesia revealed a maximum in spindle occurrence immediately after LORR with a steady decrease throughout maintenance. Immediately before RORR spindle occurrence increased again.

## Discussion

The present study summarizes substance-specific temporal and spectral EEG changes in mice during induction, maintenance, and emergence from anesthesia. It includes data from the prefrontal and primary visual cortex. For the mainstay of the analysis, we focused on the visual cortex due to huge interspecies differences in the PFC and higher correlation of primary sensory cortices [44–46].

### Sevoflurane

During induction of ether anesthesia, the second stage is marked by a “period of excitation of most major cerebral and cerebellar centers as well as reflex centers” followed by a depression of physiological functions [47]. The transient phenomenon is called paradoxical excitation and is accompanied by changes in the EEG [48]. These changes are predominantly characterized by an increase of the beta band as well as a decrease of alpha band and theta band power [49], primarily induced by GABAergic drugs [8]. In the present experiments, analogue changes were found, previously exclusively described in humans [50]. During anesthesia induction with propofol and sevoflurane, a significant increase in the beta band was recorded, while the slower theta band power and alpha band power decreased. The exact neuronal mechanisms underlying this phenomenon are still not fully understood. McCarthy et al. suggested a dosage dependent effect of GABA_A_ potentiation [51]. Small amounts can excite the postsynaptic membrane of pyramidal cells resulting in an antisynchrony of the firing of the inhibitory neurons and therefore reducing the global inhibitory effect on the network., whereas large amounts of GABAergic drugs slow postsynaptic spiking as the GABA_A_ current dominates membrane dynamics [51]. This increased inhibition results in a decreased neuronal firing rate which is reflected in the emergence of slow wave activity [52, 53].However, the mechanisms mediating paradoxical excitation do not seem to be limited to the cortex. As recently shown in vitro, propofol influences sodium channel activation and suppresses normal bursting of thalamocortical cells resulting in small chaotic oscillations that lead to irregular spiking of pyramidal cells [54]. In contrast to previous studies reporting increased frontal slow delta band and alpha band power under sevoflurane anesthesia for rats, such general EEG slowing could not be detected in the present work [55]. However, our findings are in line with human data lacking increased alpha power or low-frequency power in healthy volunteers [56]. Due to the lack of accepted anatomical definitions in frontal and prefrontal mammalian brain regions the comparability of the study results is limited [44, 45]. Additionally, the interpretation of findings is challenging considering inter-species-specific differences and a highly inconsistent nomenclature [57, 58].

### Propofol

While sevoflurane has a rather broad effect on several neurotransmitter systems, propofol acts primarily at the β-subunits of the GABA_A_ receptors [59]. Additional target sites of propofol include acetylcholine and glycine receptors [15]. Activation of the GABA_A_ receptor results in an inward chloride current leading to a hyperpolarization of postsynaptic neurons [60]. This mechanism can be observed in the inhibition of cortical pyramidal neurons [61], inhibition at the thalamic reticular nucleus resulting in reduced inputs from the thalamus to the cortex and in an enhanced inhibition at GABAergic projections from the preoptic area of the hypothalamus to the major arousal systems [19]. The present study found a significant propofol-induced increase of the alpha band power in the PFC when comparing the maintenance phase with baseline EEG. This observation partially overlaps with a phenomenon which has been described in humans as “alpha anteriorization” [62, 63]. It is characterized by an increased frontal alpha activity while the alpha power in occipital cortices dissipates. This latter part was not observed in the present study as the alpha power above the visual cortex was not influenced by propofol infusion. However, the strong occipital alpha power in the awake state seems to be a hallmark of the human brain potentially explaining the lack of a propofol-induced decrease in the occipital alpha power in mice due to a lower baseline [64]. Upon further increase of propofol dosage after LORR, frontal alpha power ceased in the present study analogue to what has been described in humans as an alpha band collapse before the occurrence of burst suppression [65]. A significant shift towards the delta band during induction or throughout the maintenance period could not be found in mice [16]. One reason could be that the experimental anesthesia was performed at the start of the lights-on period when sleep pressure is highest [66–68]. This high endogenous sleep pressure is associated with an already increased delta band power in mice prior to anesthesia [69].

### Dexmedetomidine

Dexmedetomidine exerts its sedative effects through a highly specific inhibition of norepinephrine release in the wake-promoting locus coeruleus in the brainstem via α_2_-adrenergic receptors by a G_i_-receptor coupled decrease in cAMP production, followed by an activation of K^+^-channels [70, 71]. The inhibition of the major wake-promoting pathway causes a non-rapid eye movement sleep-like neuronal signature with a general slowing of the EEG which was also observed in the present study: a significant global increase in delta power and a decrease in all other frequency bands [16, 72, 73]. This finding goes in line with findings in humans where occipital delta oscillations have been described as the most noticeable feature of dexmedetomidine induced sedation [12, 19]. More resemblances between humans and mice can be found when looking for locoregional changes. Increased frontal theta and alpha oscillations as well as intensified sleep-spindle activity in phases of light-sedation during early maintenance, correspond well with findings in humans [12, 16, 74, 75]. Sleep spindles originate from the thalamocortical network and one of their physiological function is the blockage of sensory information to the cortex thereby preventing disruptive sleep behavior [38, 74, 76]. More common patterns can be found during the recovery phase from dexmedetomidine anesthesia. The persistent occipital delta, increased theta and alpha oscillations as well as growing global beta power previously described in humans can also be observed in mice [12]. The present data also revealed discrepancies in the spectral composures of human and rodent brain activity. Dexmedetomidine typically causes an increase in occipital theta oscillations in humans [12], which was not found in mice.

### Ketamine

Ketamine mediates its effect mainly but not exclusively via a use-dependent blockage of the glutamatergic NMDA receptor [18, 77]. In sub-anesthetic concentrations ketamine is primarily blocking NMDA receptors of GABAergic, inhibitory interneurons [78]. The inhibition of inhibitory interneurons results in a disinhibition of excitatory neurons leading to an increased power in the beta and low gamma band (27–40 Hz) in humans and rodents [11, 19, 79]. Further increase of ketamine concentration during anesthesia leads to an inhibition of excitatory neurons and subsequently induced unconsciousness which is characterized by a gamma burst, transitioning into stable beta-gamma oscillations [11, 19]. Our present data from mice are in close correlation to the human data. Upon induction, a significant increase in the beta and gamma power (> 25 Hz) was revealed, while simultaneously, the activity in the alpha band decreased. During the maintenance phase, this effect persisted, before reversing during the emergence period, with beta and gamma power decreasing significantly. In contrast to human data, no initial gamma burst but a rather continuous increase in the beta and gamma band was visible in our murine study.

In summary, we found a transient beta activation during induction with sevoflurane and propofol. Propofol also induced an increase in the frontal alpha power, but neither sevoflurane nor propofol administration was associated with an increased delta activity. Dexmedetomidine infusion resulted in a general slowing of the EEG and increased spindle activity during the induction period. Ketamine, on the other hand, increased the power in the higher frequencies, but did not influence the power in the delta band.

### Limitations

Free movement of the animals during the experiments generated movement artifacts, demanding ASR-filtering. In our opinion, these artifacts are inevitable since restraining the animal would cause stress influencing the EEG as well as the anesthesia requirement [80, 81]. Additionally, the avoidance of head-fixation follows the 3 Rs as it reduces suffering of the animals.

Transferability of findings might be impaired by discrepancies between clinical application of the given substances and induction mode used in the present experiments (i.e., slow induction often is not representative of clinical practice, dexmedetomidine is not an anesthetic but a sedative). We deviated from clinical routine in order to gain the possibility to analyze the transition from wake to unconscious more detailed [82]. However, a strength of the present study is the route of application. In comparison to previous publications which mainly used an intraperitoneal injection we established a central venous access to administer the drugs intravenously – the same route of administration as used in clinical practice. Even though we used the same route, the applied doses differed hugely from clinical practice due to the high metabolic rate of mice [83]. Additionally, the drugs used in this study vary highly in their pharmacodynamic profile. While sevoflurane and propofol have only anesthetic properties, ketamine can be used as a sole agent for anesthesia inducing amnesia, analgesia, loss of consciousness and immobility [17]. Dexmedetomidine is primarily a sedative potentially explaining the multiple transitions between LORR and RORR observed in the present study. Only at very high doses of dexmedetomidine, which are not used clinically, prolonged LORR could be induced. Even though we tried to adjust for anesthetic depth by using a behavioral endpoint, the differences in the pharmacodynamic profiles of the drugs should be kept in mind when drawing conclusions from the comparison of the EEG patterns induced by the various drugs.

Apart from the general differences between rodent and human EEGs, there are technical limitations of the presented approach [84]. Whereas the literature on human findings is primarily based on scalp EEG recordings, we collected epidural EEG data. From a technical perspective, the epidural signal is not dampened by the skull and the connective tissue resulting in absolute higher amplitudes. Even though the skull might act as a low-pass filter, previous studies did not find a significant difference in the spectral composition of scalp EEG and epidural EEG signals [85, 86]. Since most of the clinically used anesthesia depth monitors only include frequencies up to 47 Hz, we also low-passed filtered the signal at 47 Hz. However, future studies should include the high gamma band especially when evaluating cognitive recovery after anesthesia [87]. Furthermore, we only evaluated changes in the frontal and the visual cortex. We focused on these areas since the electrodes for anesthesia brain function monitoring devices are usually placed frontally and the primary sensory cortices like the visual cortex were suggested to be well preserved between species [46]. However previous studies demonstrated that, different anesthetics have specific effects on different cortical areas [56, 63]. For this reason, a high-density EEG to evaluate drug specific effects on distinct cortical areas would be a great approach for subsequent studies.

## Conclusions

The present study demonstrated substance-specific EEG patterns in mice. The observed changes in power distribution induced by intravenous and inhalational anesthetic agents were similar to previous descriptions in human EEGs, but also included significant differences especially in the lower frequencies. It provides the basis to reveal fundamental neurophysiological principles of anesthesia actions at cortical and subcortical level.

### Electronic supplementary material

Below is the link to the electronic supplementary material.

Supplementary Material 1

## Data Availability

The datasets used and analyzed during the current study are available from the corresponding author on reasonable request.
